# Suppression of PC-1/PrLZ sensitizes prostate cancer cells to ionizing radiation by attenuating DNA damage repair and inducing autophagic cell death

**DOI:** 10.18632/oncotarget.11470

**Published:** 2016-08-22

**Authors:** Zeng-Fu Shang, Qiang Wei, Lan Yu, Fang Huang, Bei-Bei Xiao, Hongtao Wang, Man Song, Li Wang, Jianguang Zhou, Jian Wang, Shanhu Li

**Affiliations:** ^1^ School of Radiation Medicine and Protection, Medical College of Soochow University, Collaborative Innovation Center of Radiation Medicine of Jiangsu Higher Education Institutions, Suzhou, Jiangsu 215123, China; ^2^ Laboratory of Medical Molecular Biology, Beijing Institute of Biotechnology, Beijing 100850, China; ^3^ Department of Urology, Nanfang Hospital, Southern Medical University, Guangzhou, Guangdong, 510515, China

**Keywords:** PC-1/PrLZ, PCa, autophage, radiotherapy

## Abstract

Radiotherapy is promising and effective for treating prostate cancer but the addition of a tumor cell radiosensitizer would improve therapeutic outcomes. PC-1/PrLZ, a TPD52 protein family member is frequently upregulated in advanced prostate cancer cells and may be a biomarker of aggressive prostate cancer. Therefore, we investigated the potential role of PC-1/PrLZ for increasing radioresistance in human prostate cancer cell lines. Growth curves and survival assays after g-ray irradiation confirmed that depletion of endogenous PC-1/PrLZ significantly increased prostate cancer cell radiosensitivity. Irradiation (IR) increased PC-1/PrLZ expression in a dose- and time-dependent manner and increased radiosensitivity in PC-1/PrLZ-suppressed cells was partially due to decreased DNA double strand break (DBS) repair which was measured with comet and gH2AX foci assays. Furthermore, depletion of PC-1/PrLZ impaired the IR-induced G2/M checkpoint, which has been reported to be correlate with radioresistance in cancer cells. PC-1/PrLZ-deficient cells exhibited higher level of autophagy when compared with control cells. Thus, specific inhibition of PC-1/PrLZ might provide a novel therapeutic strategy for radiosensitizing prostate cancer cells.

## INTRODUCTION

Prostate cancer is the most common cancer and the second leading cause of death for men in Europe and in the US [1, 2]. Also, prostate cancer is increasing in Asia [3]. Approximately 27,000 deaths were attributable to prostate cancer in the US in 2009, representing 10% of all cancer-related deaths [1]. Radiotherapy is effective and common for treating prostate cancer and offers some survival advantages for localized stages of prostate cancer [1]. However, cancer cell resistance to radiation and severe side effects from irradiation of adjacent normal tissues limits clinical applications radiotherapy success. Thus, many prostate cancers with radiotherapy will eventually fail and develop metastatic disease in less than 5 years [4]. Therefore, radiotherapy requires study to better understand mechanistic pathways involved in the establishment and progression of prostate cancer radioresistance. Then, identifying novel targets to enhance the killing effect of irradiation against cancer cells and to decrease the side effect of irradiation on normal tissue is still an important strategy in the treatment of prostate cancers.

LNCaP cells are androgen-responsive, non-metastatic, and marginally tumorigenic prostate cancer cells and C4-2 cells are a subline derived from LNCaP cells that have a more aggressive phenotype including androgen independence and osseous metastases [5]. LNCaP and C4-2 cells are frequently to model prostate cancer and investigate mechanisms of prostate cancer progression. Our previous work indicates that C4-2 cells are more likely to acquire radio-resistance than parental LNCaP cells [6]. Previous studies indicate that the human prostate and colon gene-1 (*PC-1*, also known as *PrLZ*) is over-expressed in C4-2 cells compared with LNCaP cells according to cDNA microarray analysis [7]. PC-1/PrLZ is a member of the TPD52 protein family which includes members highly associated with proliferation and progression of various cancers, including breast, lung and prostate adenomas [8–10]. Most importantly, PC-1/PrLZ is specifically expressed in prostate tissues, whereas other TPD52 family members are uniformly expressed in different tissues [7]. The *PC-1/PrLZ* gene is located at chromosome 8q21.1, the locus most frequently amplified in human prostate cancers [7]. As expected, the *PC-1/PrLZ* gene is amplified in many prostate cancer cases as evidenced by fluorescence in situ hybridization (FISH) analysis with a PC-1/PrLZ-specific probe. Moreover, recent evidence indicates that PC-1/PrLZ is frequently overexpressed in advanced prostate cancer tissues [11], and this increased expression contributes to malignant phenotypes, including androgen-dependent and-independent growth, anchor-independent growth and tumorigenicity [12, 13]. These reports suggest that PC-1/PrLZ possesses oncogenic characteristics and is highly associated with malignant progression in prostate cancer.

To understand whether PC-1/PrLZ is important to radio-resistance in prostate cancer cells, gain-of-function and loss-of-function analyses were performed to elucidate the functional significance and the related mechanism of PC-1/PrLZ in prostate cancer cells after ionizing radiation (IR). Here, we report that PC-1/PrLZ conferred radio-resistance to prostate cancer cells and suppression of PC-1/PrLZ reduced cell repair of DNA double-strand breaks (DSBs) and attenuated activation of the G2 checkpoint. Moreover, suppression of endogenous PC-1/PrLZ radiosensitized prostate cancer cells, contributing to increased induction of autophagic cell death but not apoptosis and senescence after IR. Thus, PC-1/PrLZ is a novel candidate involved in DNA DSB repair and radioresistance, and targeting PC-1/PrLZ may offer promise for an effective method for enhancing the efficiency of radiation therapy for prostate cancer.

## RESULTS

### PC-1/PrLZ expression was induced by IR in prostate cancer cells

**Figure 1:**
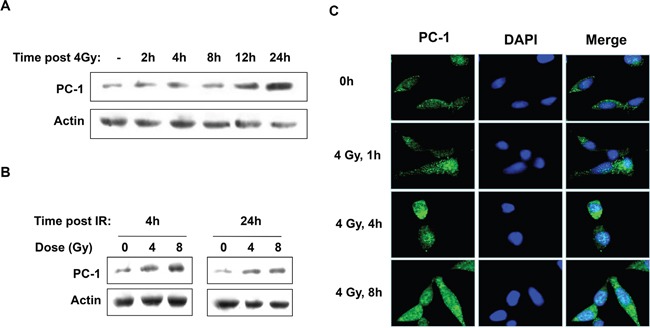
IR upregulated PC-1/PrLZ expression in prostate cancer cells **A.** PC-1/PrLZ expression was measured at different time points after 4-Gy irradiation. **B.** Dose-dependent upregulation of PC-1/PrLZ expression 4 and 24 h post-irradiation. **C.** C4-2 cells were fixed at different time points after 4-Gy irradiation and stained with PC-1/PrLZ antibody. Images show that PC-1/PrLZ expression was enhanced after 4-Gy irradiation.

To determine the association between PC-1/PrLZ and the cellular response to radiation, expression and localization of PC-1/PrLZ in prostate cancer cells after irradiation were measured. Figure 1A, 1B and Supplementary Figure S1 show that PC-1/PrLZ expression increased in C4-2 and C4-2B cells after IR, and radiation-induced expression persisted for at least 24 h after 4-Gy irradiation (Figure 1A and Supplementary Figure S1). IR increased PC-1/PrLZ expression in a dose-dependent manner (Figure 1B) and immunofluorescent staining analysis revealed that endogenous PC-1/PrLZ localized predominantly in the cytoplasm and faintly in the nuclei of C4-2 cells (Figure 1C). However, 4-Gy irradiation partially increased nuclear localization of PC-1/PrLZ. Immunofluorescence also indicated increased expression of PC-1/PrLZ at 4 and 8 h after 4-Gy irradiation.

### PC-1/PrLZ expression is correlated with radioresistance in prostate cancer cells

**Figure 2:**
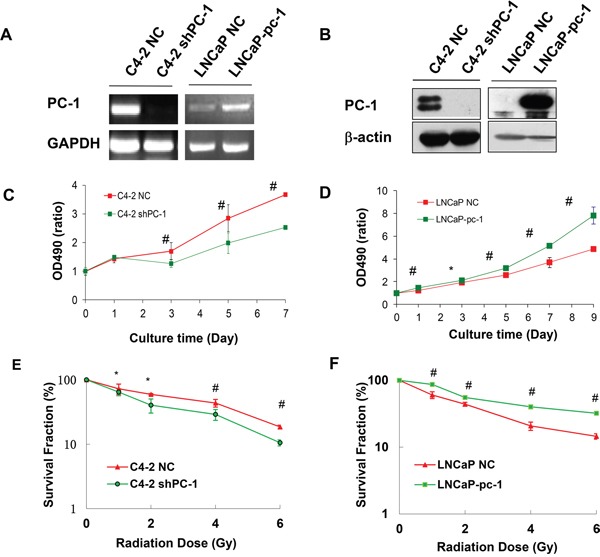
Decreased PC-1/PrLZ expression sensitized prostate cancer cells to IR; PC-1/PrLZ overexpression increased radioresistance **A.** RT-PCR was used to measure PC-1/PrLZ mRNA expression in PC-1/PrLZ-suppressed C4-2 shPC-1 cells, PC-1/PrLZ-overexpressing LNCaP-pc-1 cells and paired controls, C4-2 NC cells and LNCaP-NC cells, respectively. **B.** PC-1/PrLZ protein was measured in PC-1/PrLZ-suppressed C4-2 shPC-1 cells, PC-1/PrLZ-overexpressing LNCaP-pc-1 cells, and paired controls, C4-2 NC cells and LNCaP-NC cells, respectively. **C.** C4-2 shPC-1 cell and control C4-2 NC cell proliferation was measured at different times after 4-Gy irradiation. Data are means ± standard deviations from three independent experiments. **D.** LNCaP-pc-1 cell and control LNCaP-NC cell proliferation was measured at different times after 4-Gy irradiation. Data are means ± standard deviations from three independent experiments. **E.** Survival curves of PC-1/PrLZ-suppressed C4-2 shPC-1 cells and control C4-2 NC cells after irradiation (0-6 Gy). Data are means ± standard deviations from three independent experiments. **F.** Survival curves of PC-1/PrLZ-overexpressing LNCaP-pc-1 cells and control LNCaP-NC cells after irradiation (0-6 Gy). Data are means ± standard deviations from three independent experiments. * *P* < 0.05, # *P* < 0.01.

To examine the effect of PC-1/PrLZ on prostate cancer cell radiosensitivity, we knocked down endogenous *PC-1/PrLZ* with shRNA in C4-2 cells expressing high levels of PC-1/PrLZ. In addition, we stably transfected and expressed the exogenous *PC-1/PrLZ* gene in the PC-1/PrLZ-hypo-expressing cell line LNCaP. Both RT-PCR (Figure 2A) and Western blot (Figure 2B) confirmed that PC-1/PrLZ expression was suppressed in C4-2 shPC-1 cells and increased in LNCaP-pc-1 cells compared with C4-2 NC cells and LNCaP-NC cells, respectively. MTT assay (Figure 2C) and a clonogenic assay (Figure 2E) confirmed that shRNA-mediated suppression of PC-1/PrLZ expression (C4-2 shPC-1) significantly sensitized C4-2 cells to IR. In contrast, overexpression of PC-1/PrLZ in LNCaP (LNCaP-pc-1) cells significantly increased radioresistance of LNCaP cells (Figure 2D and 2F). The surviving fraction (SF) at 2Gy (SF_2_) for C4-2 cells was reduced from 59.3%±1.9% to 40.4%±10% when we knockdown endogenous *PC-1* expression, and the SF2 of LNCaP was increased from 43.9%±3% to 55.3%±3.2% when we overexpressed *PC-1* gene in it, suggesting PC-1 increased radioresistance in prostate cancer cells. (Figure 2E and 2F)

### Suppression of PC-1/PrLZ decreased DNA DBS repair capacity which induced the prolonged activation of the DNA damage response signal pathway

**Figure 3:**
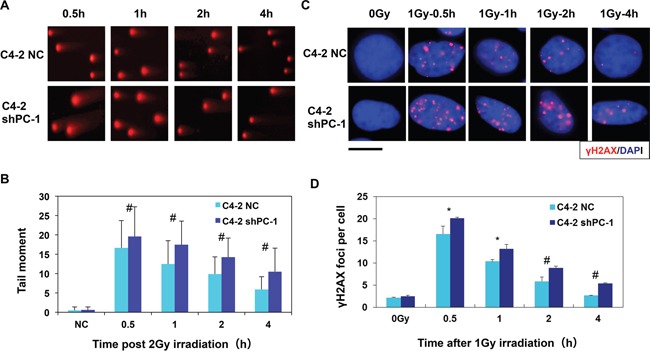
Decreased capacity of DNA DBS repair in PC-1/PrLZ-knockdown cells **A.** Comet images of DNA DSBs detected with neutral single cell gel electrophoresis at 0.5-4 h after irradiation. (average, more than 100 cells were counted, three independent assays) **B.** Repair kinetics of 4-Gy-induced DNA DSBs were measured. **C.** C4-2 shPC-1 and C4-2 NC cells were irradiated with 2-Gy γ-rays and immunostained for phospho-γH2AX (red) foci 0.5 h to 4 h after irradiation (The scale is 10 μM). **D.** Residual γ-H2AX foci per nucleus at the indicated time points after irradiation (average, 50 nuclei were counted, three independent assays). *P < 0.05, #*P* < 0.01.

Neutral single-cell gel electrophoresis assay (comet assay) was used to detect DNA DSBs damage in C4-2 shPC-1 and C4-2 NC cells after 4-Gy irradiation. Comet tails of C4-2 shPC-1 cells were longer than those in C4-2 NC control cells at 0.5 to 4 h post-irradiation (Figures 3A and 3B). Next, the phosphorylated H2AX (γH2AX) foci assay is a sensitive method for measuring DNA DSBs. C4-2 shPC-1 and C4-2 NC cells were immunofluorescently stained for γH2AX (red) foci, and DSB repair kinetics were assessed by counting foci per cell. C4-2 shPC-1 cells had slower DSBs repair compared with controls at 0.5 to 4 h post-irradiation (Figures 3C and 3D).

**Figure 4:**
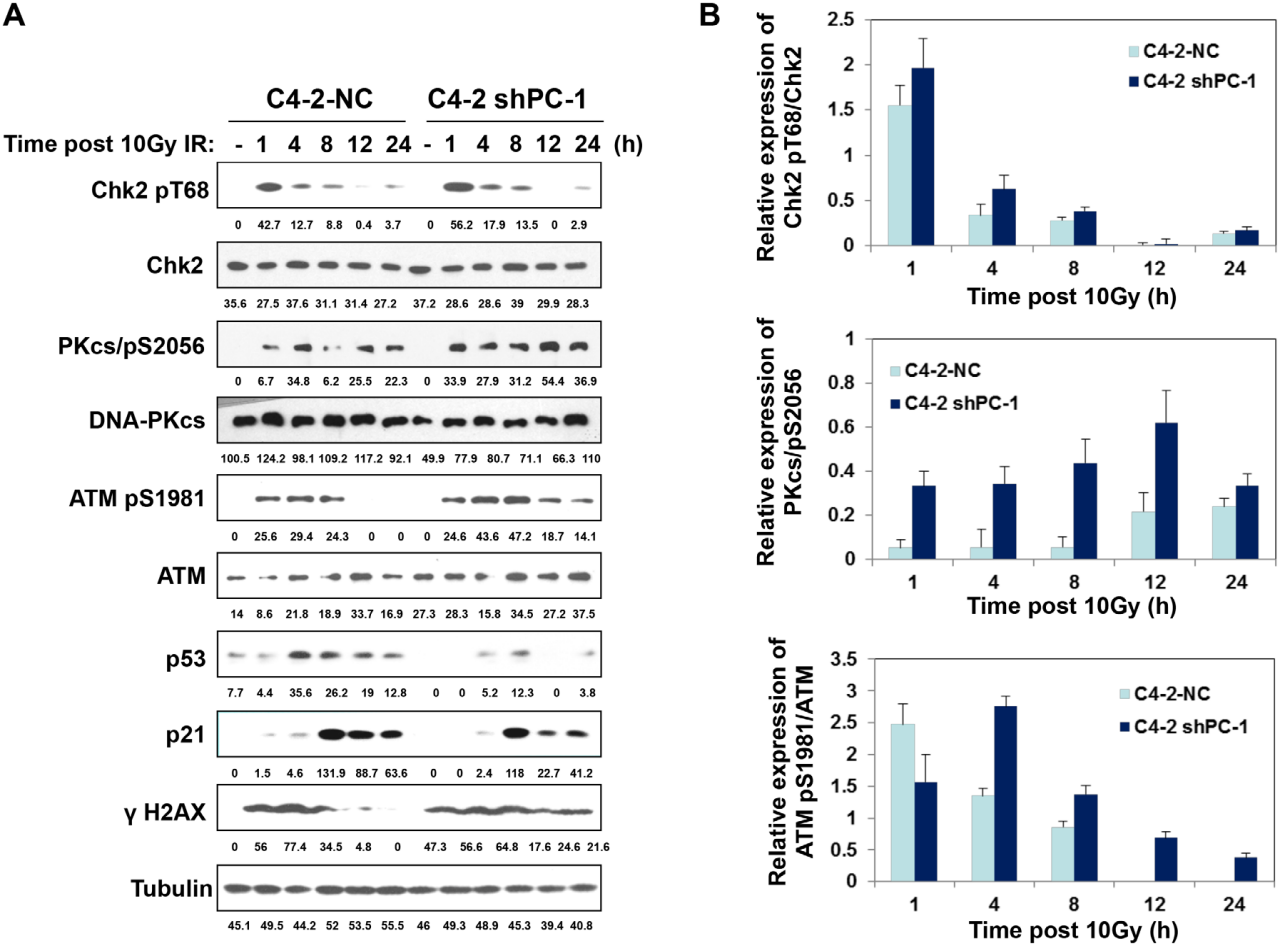
Radiation-induced phosphorylation of DNA-PKcs and ATM **A.** C4-2 NC and C4-2 shPC-1 cells were irradiated with 10-Gy γ-ray or were mock irradiated. ATM, DNA-PKcs, Chk2, p21 and p53 protein and phosphorylation were measured. **B.** Relative expression of phosphorylated Chk2, ATM and DNA-PKcs to total Chk2, ATM and DNA-PKcs proteins after IR were quantified from three independent experiments as shown in (a). Columns, mean percentage of three independent experiments.

DNA-PKcs and ATM are two key enzymes of nonhomologous end joining (NHEJ) repair, so we measured phosphorylation of these enzymes at specific sites that critical to DNA-PKcs and ATM repair activity with Western blot. Figure 4 shows that suppression of PC-1/PrLZ expression markedly prolonged phosphorylation of DNA-PKcs and ATM at S2056 and S1981 respectively in C4-2 prostate cancer cells after 10-Gy irradiation. Consistent with these data, phosphorylation of Chk2, which localized downstream of DNA-PKcs and ATM kinases and was important to DNA damage-induced cell cycle checkpoint activation, was also enhanced in PC-1/PrLZ deficient prostate cancer cells. Thus, PC-1/PrLZ-deficient C4-2 shPC-1 cells are more sensitive to irradiation and this may be partially attributed to prolonged DNA damage repair progression.

### Depletion of PC-1/PrLZ impaired irradiation-induced G2/M cell cycle checkpoint

**Figure 5:**
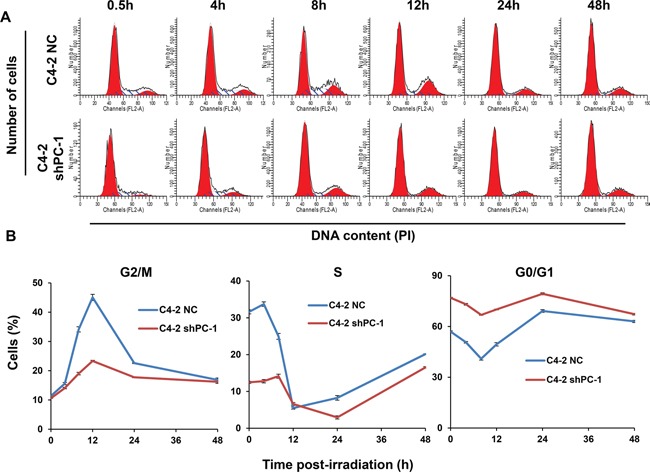
Cell cycle distribution after irradiation **A.** Representative histograms of flow cytometry of C4-2 NC and C4-2 shPC-1 cells at different times after 4-Gy γ-ray irradiation. **B.** Quantitative data of proportion of cells in G2/M, S or G0/G1 phases at different times after irradiation. Data are means ± standard deviations from three independent experiments.

In mammalian cells, IR halts cell cycle progression at G1, S and G2 phases and these cell cycle checkpoints provide cells with sufficient time to repair damaged DNA. We show that G2/M arrest was evident in prostate cancer cells as early as 4 h after 4-Gy irradiation, and peaked after ~12 h (Figures 5A and 5B). Importantly, the proportion of cells arrested at the G2/M phase in *PC-1/PrLZ*-depleted cells (C4-2 shPC-1) was dramatically less than in controls (C4-2 NC), indicating that PC-1/PrLZ participates in the IR-induced G2/M checkpoint. Although we observed robust activation of checkpoint kinase 2 (Chk2) in PC-1/PrLZ-deficient cells, the other signaling pathway might be involved in G2/M checkpoint maintenance, such as p53-p21. Thus, we suggest that loss of PC-1/PrLZ decreased expression of p53 and p21 in C4-2 cells after exposure to IR (Figure 4A).

### Radiosensitization by suppressing PC-1/PrLZ is attributed to induction of enhanced autophagic cell death

Apoptotic induction was measured in PC-1/PrLZ-deficient C4-2 and control cells after IR and Supplementary Figure S2 shows stained with α-tubulin antibody (green) and cleaved caspase-3 antibody (red), which is a molecular marker of apoptosis. In addition, DNA was quantified with DAPI. In both PC-1/PrLZ-deficient C4-2 and control cells, few apoptotic cells were evident and there was a limited increase in apoptotic cells 3 days after 4-Gy irradiation. Furthermore, loss of PC-1/PrLZ did not promote IR-induced senescence (Supplementary Figure S3). Therefore, apoptosis and senescence is not the primary mechanism by which cell death is induced after irradiation in these two cell sublines, especially in PC-1/PrLZ-deficient C4-2 cells.

**Figure 6:**
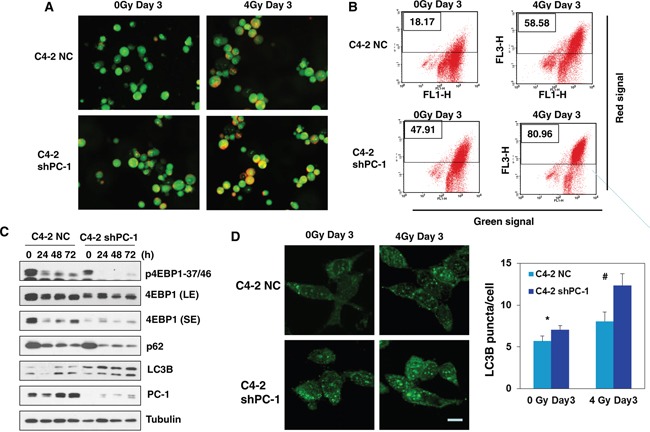
Suppression of PC-1/PrLZ induced autophagy in C4-2 cells after IR treatment **A.** Cells were grown in the presence/absence of 4-Gy irradiation for 3 days, and fluorescent images of acridine orange-stained AVO-positive cells are shown. **B.** Flow cytometry was used to measure autophagy (cells treated as depicted in A). **C.** LC3B, 4E-BP1, P62, PC-1 protein and phosphorylation was measured at the indicated time points after 4-Gy irradiation. **D.** Immunofluorescence (IF) depicting puncta of LC3 and mean LC3 puncta in C4-2 NC and C4-2 shPC-1 cells at indicated time points after 4-Gy irradiation. Data are means ± standard deviations from three independent experiments. (#*p*< 0.01 compared with controls).

Recently, several studies have suggested that autophagy, which is recognized as a survival mechanism in response to metabolic stress, can also mediate the pro-death process during stressful conditions such as irradiation. Our previous work indicated that PC-1/PrLZ blocks autophagy by maintaining protein stability of 4E-BP1 [14]. To test whether the effect of PC-1/PrLZ in irradiated cells depended on induction of autophagic cell death, we analyzed the formation of acidic vesicular organelles (AVO) using acridine orange staining with fluorescent imaging and flow cytometry. Acridine orange-positive cells were bright red. As expected, 4-Gy irradiation increased autophagic cells, and PC-1/PrLZ-deficient C4-2 cells had more acridine orange-positive staining compared with controls, especially after irradiation (Figures 6A and 6B). After 4-Gy irradiation, acridine orange-positive cells increased from 18.17% to 58.58% for C4-2 NC cells and 47.91% to 80.96% for C4-2 sh cells. In addition, increased expression of LC3B was observed, which is a hallmark of autophagy, and p62 protein decreased in PC-1/PrLZ-knockdown C4-2 shPC-1 cells compared with controls after 4-Gy irradiation (Figure 6C). Our previous work indicated that PC-1/PrLZ maintains protein stability of 4E-BP1, allowing PC-1/PrLZ overexpressing cells overcome rapamycin-induced autophagy. Here, we report that irradiation suppressed 4E-BP1 phosphorylation and protein in C4-2 cells (Figure 6C). Depletion of PC-1/PrLZ enhanced 4E-BP1 inhibition in response to IR (Figure 6C). We also measured LC3 granules with immunostaining with LC3 antibody and data support results depicted in Figure 6D. These observations suggest that suppression of PC-1/PrLZ expression may radiosensitize prostate cancer cells by inducing autophagic cell death.

## DISCUSSION

PC-1/PrLZ contains a distinctive domain similar to other TPD52 family members in the N-terminus. Therefore, we designed a shRNA against a PC-1/PrLZ-specific domain located at the NH2-terminus, which effectively suppressed expression of PC-1/PrLZ but not TPD52 (data not shown). In the present study, we report that the loss of PC-1/PrLZ expression sensitized prostate cancer cells to IR and perturbed DNA DSB repair. Importantly, we demonstrated that IR induced expression of PC-1/PrLZ. Thus, PC-1/PrLZ contributes DNA damage repair and response.

DNA DSBs are important DNA lesions that promote cell death in response to IR and these are repaired via two processes: NHEJ and homologous recombination (HR) [15]. NHEJ is the predominant process of DNA DSB repair. Ataxia-telangiectasia mutated (ATM) and DNA-PKcs are two major members of the phosphatidylinositol 3-kinase-like family, which play a dominant role in the NHEJ repair pathway. ATM is associated with cell cycle checkpoint regulation and the DNA damage response after IR-induced DSBs [16]. In contrast, DNA-PKcs plays a proximal role in NHEJ, triggering DSB-induced apoptosis and DNA damage-related mitotic catastrophe [17–20]. DNA-PKcs has serine/threonine kinase activity, and activated DNA-PKcs phosphorylates downstream targets, including Artemis, XRCC4, p53, MDM2, c-Abl, and DNA-PKcs itself after IR-induced DNA DSB formation [21]. Recently, Goodwin and colleagues revealed a positive feedback circuit between DNA-PKcs and the androgen receptor (AR) signal pathway after IR in prostate cancer cells [22]. Studies from the same group show that DNA-PKcs exists in the same complex with AR and facilitates AR activation in castration-resistant prostate cancer (CRPC) [23]. As a novel oncogenic protein, PC-1/PrLZ has also been shown to enhance AR transcription activity via promoting AR nuclear translocation and blocking AR degradation [24]. Consistent with these reports, we observed decreased basal DNA-PKcs protein in PC-1/PrLZ-deficient C4-2 cells compared to controls (Figure 4). We also found that IR promoted nuclear translocation of PC-1/PrLZ. The nuclear localization of PC-1/PrLZ might further contribute to AR-mediated DNA-PKcs transcription after irradiation. However, IR-induced phosphorylation of DNA-PKcs at the Ser2056 site was significantly enhanced in PC-1/PrLZ-knockdown cells compared with control, suggesting prolonged DNA damage repair kinetic mediated by loss of PC-1/PrLZ might lead to compensatory activation of DNA-PKcs (Figure 4). TPD52 was recognized as a radiosensitizer via negatively regulated ATM and our findings confirmed that loss of PC-1/PrLZ did not affect ATM protein but promoted phosphorylation of ATM in response to IR, which might also occur after DNA repair impairment (Figure 4). Previous studies have indicated that TPD52 transcripts were dramatically downregulated in cancer patient blood cancer after radiotherapy [8, 25]. These investigations suggest distinctive roles for PC-1/PrLZ and TPD52 in DNA damage response but how the N-terminus-specific region of PC-1/PrLZ confers unique different function in this process warrants further investigation.

Arrest at the G2/M cell cycle checkpoint is a major cellular response to DNA damage that provides sufficient time for DSB repair and prevents cells with damaged DNA from dividing [26, 27]. We compared cell cycle changes induced by IR between PC-1/PrLZ-deficient and control cells and noted a weakened G2 arrest in PC-1/PrLZ-silenced C4-2 cells after IR compared with controls (Figure 5). The G2/M checkpoint was beneficial to DNA damage repair and was critical for preventing cell death, suggesting that PC-1/PrLZ might contribute to prostate cancer cell radioresistance by inducing a robust G2/M checkpoint. In contrast, prolonged G2/M arrest contributed to IR-mediated apoptosis as measured with immunofluorescence and antibodies against cleaved-caspase-3, a hallmark of apoptosis. Apoptosis increased in both PC-1/PrLZ-deficient and control prostate cancer cells, and apoptosis was greater in controls compared with PC-1/PrLZ-deficient cells. Therefore, other types of cell death sensitize PC-1/PrLZ-deficient prostate cancer cells to IR.

Data indicate that IR induces autophagy in various cancer cells independent of apoptosis [28, 29]. Autophagy is a lysosomal degradation pathway that eliminates damaged or potentially dangerous proteins and organelles under adverse conditions to protect cells from metabolic stress [30, 31]. However, previous studies suggest that autophagy also functions as a pro-death mechanism that is frequently activated in tumor cells treated with chemotherapy or radiotherapy [28, 29]. We quantified the accumulation of AVOs after IR using acridine orange staining [32] and observed that both IR and PC-1/PrLZ silencing induced autophagy. Treatment of PC-1/PrLZ-deficient cells with IR increased AVOs and cleaved LC3B compared to control cells. PTEN is a novel tumor suppressor that balances proliferation, survival, and apoptosis by suppressing the phosphatidylinositol-kinase/Akt pathway. Loss of PTEN is a common phenomenon in aggressive prostate cancer (approximately 70% of aggressive prostate cancer cases) [33–35]. Therefore, the mammalian target of rapamycin (mTOR) is frequently hyper-activated in prostate cancer. Recent research has shown that mTOR inhibition using the specific inhibitor RAD001 increased radiosensitivity of prostate cancer cells by inducing autophagic cell death [36]. Our recent work confirmed that PC-1/PrLZ interacts with 4E-BP1, a downstream factor of mTOR, and mains its protein stability via blocking ubiquitin-mediated proteasome degradation, through which PC-1/PrLZ inhibits autophagy and contributes to chemoresistance of prostate cancer cells [14]. As shown in Figure 6C, 4E-BP1 was significantly downregulated in PC-1/PrLZ-depleted C4-2 cells, and this is consistent with our previous conclusions. Furthermore, irradiation inhibited phosphorylation and expression of 4E-BP1 in prostate cancer cells, an outcome similar to previous observations in breast cancer cells by Tofilon' group [37]. Interestingly, Dubois and colleagues proved that 4E-BP1 knockdown sensitized glioblastoma xenograft tumors to IR [38]. Moreover, depletion of 4E-BP1 facilitates chemosensitization via inducing autophagic cell death in prostate cancer cells [39]. Work by our laboratory and that of others revealed that both PC-1/PrLZ and 4E-BP1 were overexpressed in prostate cancers [14, 40], therefore, the PC-1/PrLZ-4E-BP1 signaling pathway represents a logical therapeutic target to increase tumor cell radio- and chemo-sensitivity. The exact role of PC-1/PrLZ-4E-BP1 in regulating autophagy progression will require additional studies. Leontieva and colleagues found that inhibition of mTORC1/C2 activities blocks cell cycle arrest induced senescence [41]. Our present study revealed that PrLZ/PC-1 didn't increase IR induced senescence in prostate cancer cells (Supplementary Figure S3), suggesting PrLZ/PC-1 might did not regulate other mTOR signal downstream targets which were correlated with senescence regulation. In summary, our findings provide new insight into the role of PC-1/PrLZ for promoting DNA DSB repair and inhibiting IR-induced autophagic cell death and suggest that PC-1/PrLZ may be a novel radio-therapeutic target for treating advanced prostate cancer.

## MATERIALS AND METHODS

### Cell culture and irradiation

The prostate cancer cell lines LNCaP and C4-2 were grown in RPMI 1640 (Invitrogen, Carlsbad, CA, USA) supplemented with 8% fetal bovine serum (HyClone), 10 mM HEPES, and 1 mM sodium bicarbonate in a humidified incubator at 37°C with 5% CO_2_. LNCaP-zero and LNCaP-pc-1 cells were generated from LNCaP cells as previously described [13]. We stably transfected C4-2 cells with a PC-1-specific shRNA construct targeting the *PC-1/PrLZ* gene (AAGCTATCTCTACTTGTCTCC) or a negative control (NC) shRNA construct to generate C4-2 sh and C4-2 NC cells, respectively. The irradiation was performed using a cobalt-60 γ-ray source at a dose rate of 1.74 Gy/min at room temperature.

### Antibodies and chemicals

The antibody against 46 amino acid residues at the N-terminus of PC-1 was generated by our laboratory [13]. All of the other antibodies were purchased commercially: anti-DNA-PKcs total (Santa Cruz, CA, USA), anti-phosphorylated DNA-PKcs (Ser2056; Abcam, UK), anti-cleaved caspase-3, anti-LC3B, anti-ATM total, anti-pATM (Ser1981), anti-Chk2 total, anti-pChk2 (Thr68), anti-p21, anti-4E-BP1 (Cell Signaling, Beverly, MA), anti-α-tubulin (St. Louis, MO, USA) and anti-γH2AX (Ser139; Upstate Biotechnology, Charlottesville, VA) antibodies. Secondary antibodies were horseradish peroxidase (HRP)-conjugated anti-rabbit IgG (H+L) or HRP-conjugated anti-mouse IgG (H+L) purchased from Zhongshan Golden Bridge Biotechnology.

### Clonogenic survival

The cells were diluted and plated into 60-mm Petri dishes immediately after exposure to increasing doses (0, 1, 2, 4 and 6 Gy) of γ-ray irradiation. After 10 days of incubation, the colonies were fixed using methanol and stained with Giemsa solution. After counting colonies of more than 50 cells, the survival rates were calculated, and the survival curves were plotted.

### 3-(4, 5-dimethylthiazol-2-yl)-2, 5-diphenyltetrazolium bromide (MTT) assay

Cell proliferation was assessed using the 3-(4, 5-dimethylthiazol-2-yl)-2, 5-diphenyltetrazolium bromide (MTT) assay. In total, 2,000 to 3,000 cells were seeded in 96-well plates with 100 μl of medium and irradiated with 4 Gy of γ-rays 12 hours later. The cell number was determined on the indicated day after irradiation. In total, 20 μl of MTT reagent (2.5 mg/ml, diluted in PBS, Amresco, NJ, USA) was added to the medium, and the cells were incubated for 4 hours at 37°C. The formazan crystals were dissolved in 150 μl of DMSO. The absorbance was measured at a wavelength of 490 nm.

### The detection of γH2AX foci

The phosphorylation of H2AX (γH2AX) was used as an indicator of DNA DSB. The cells were cultured for the indicated times to repair DNA lesions after 1 Gy of irradiation. The cells were fixed in 4% paraformaldehyde/PBS for 30 min, permeabilized in 0.5% Triton X-100/PBS for 15 min, and blocked in 1% bovine serum albumin for 30 min. The samples were incubated with anti-γH2AX antibodies (1:500) for 1 hour, washed in PBS for 10 min three times, and incubated with TRITC-conjugated goat anti-mouse secondary antibodies (1:400) for 1 h. The cells were washed for 10 min three times and mounted using VECTASHIELD mounting medium with 4′, 6-diamidino-2-phenylindole (DAPI) (Vector Laboratories, Burlingame, CA). The number of γH2AX foci was examined using a fluorescence microscope.

### Comet assay for DNA double-strand breaks

The cells were plated onto 60-mm dishes, allowed to attach, and irradiated with 4 Gy of γ-rays. After treatment, the cells were collected and mixed with low melting point (LMP) agarose at 37°C and then spread onto a Comet assay slide. The slides were left to dry at 4°C, dipped in neutral lysis solution and subjected to electrophoresis. The slides were gently washed with neutralization buffer and stained with ethidium bromide before being visualized and analyzed under a fluorescence microscope. The tail moments [%DNA in the tail × tail length (μm)] were used as a measure of DNA damage.

### Flow cytometric analysis of the cell cycle

C4-2 NC and C4-2 shPC-1 cells were harvested and fixed using 75% ethanol either immediately or at the indicated time after 4 Gy of γ-ray irradiation. The cells were resuspended in PBS containing 0.1% saponin and 1 μg/ml RNase A (Sigma, St. Louis, MO, USA), incubated for 20 minutes at 37°C, and stained with 25 μg/ml propidium iodide (PI) (Sigma). The cell cycle distribution was analyzed using flow cytometry, and more than 10,000 cells per sample were counted.

### Immunofluorescence microscopy

C4-2, C4-2 NC and C4-2 shPC-1 cells were plated in poly-D-lysine-coated culture slides (BD Pharmingen), washed in PBS, fixed in 4% paraformaldehyde/PBS for 30 min, permeabilized using 0.5% Triton X-100/PBS for 15 min, and blocked in 1% bovine serum albumin for 30 min. Immunostaining was performed using anti-PC-1/PrLZ, anti-α-tubulin and anti-cleaved caspase-3 antibodies for 2 hours at room temperature. After three 10-min washes, the cells were stained with anti-mouse/rabbit rhodamine-conjugated (1:200) and anti-mouse/rabbit FITC-conjugated (1:200) secondary antibodies. DNA was stained using 4′, 6-diamidino-2-phenylindole (DAPI) in mounting solution. Confocal immunofluorescence microscopy was performed using an LSM 510 laser-scanning confocal microscope (Zeiss). The expression level of PC-1 after 4-Gy irradiation was quantified at the indicated times using Image-Pro Plus software. Cleaved caspase-3-positive cells (red) with condensed and fragmented DNA were characteristic of apoptosis. Three independent experiments were performed.

### Detection of acidic vesicular organelles

The cells were grown in 6-well plates and allowed to attach overnight. Cells were harvested and incubated with 1 μg/ml acridine orange/PBS for 15 min. The cell number is around 10×10^5^/ml. Then cells were washed and suspended with PBS. The cells were dropped on the slider and examined under a fluorescence microscope (Olympus) at ×40 magnification 3 days after 4 Gy of γ-ray irradiation. Untreated cells were also cultured for 3 days as a negative control. The samples were collected for FACScan and analyzed using ModFitLT software to quantify cells that were positive for acidic vesicular organelles.

### Statistical analysis

Statistical calculations were performed using SPSS 13.0. The data in this study were presented as means ± standard deviation (sd). Student's t test and x2 test were used when appropriated. P < 0.05 was judged to be statistically significant.

## SUPPLEMENTARY FIGURES



## Abbreviations

PC-1/PrLZ: human prostate and colon gene-1
IR: ionizing radiation
DSBs: DNA double-strand breaks
mTOR: mammalian target of rapamycin
4E-BP1: eIF4E-binding protein 1
