# Supplementary material for: Suppression of PC-1/PrLZ sensitizes prostate cancer cells to ionizing radiation by attenuating DNA damage repair and inducing autophagic cell death
Source: Oncotarget. 2016 Aug 22;7(38):62340–51. doi: 10.18632/oncotarget.11470 (PMC5308731; doi:10.18632/oncotarget.11470)
Supplement: Supplementary file 1 [file oncotarget-07-62340-s001.pdf]

Suppression of PC-1/PrLZ sensitizes prostate cancer cells to ionizing radiation by attenuating DNA damage repair and inducing autophagic cell death

SUPPLEMENTARY FIGURES

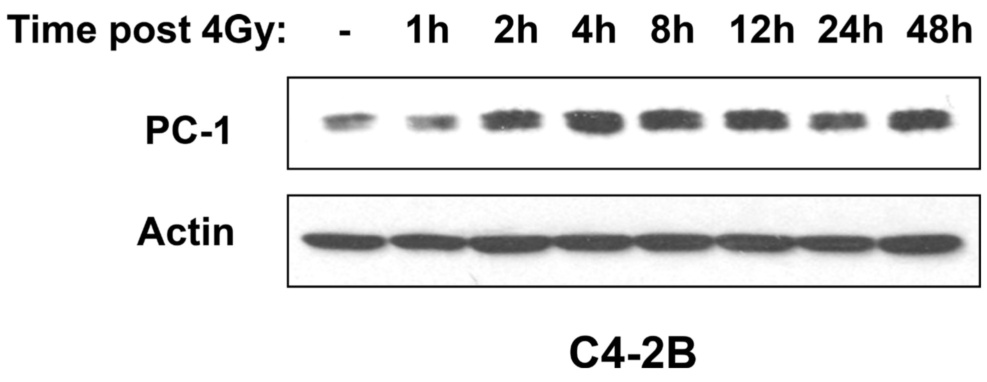

Supplementary Figure S1: IR upregulated PC-1/PrLZ expression in prostate cancer cells. A. PC-1/PrLZ expression was measured in prostate cancer C4-2B cells at different time points after 4-Gy irradiation.

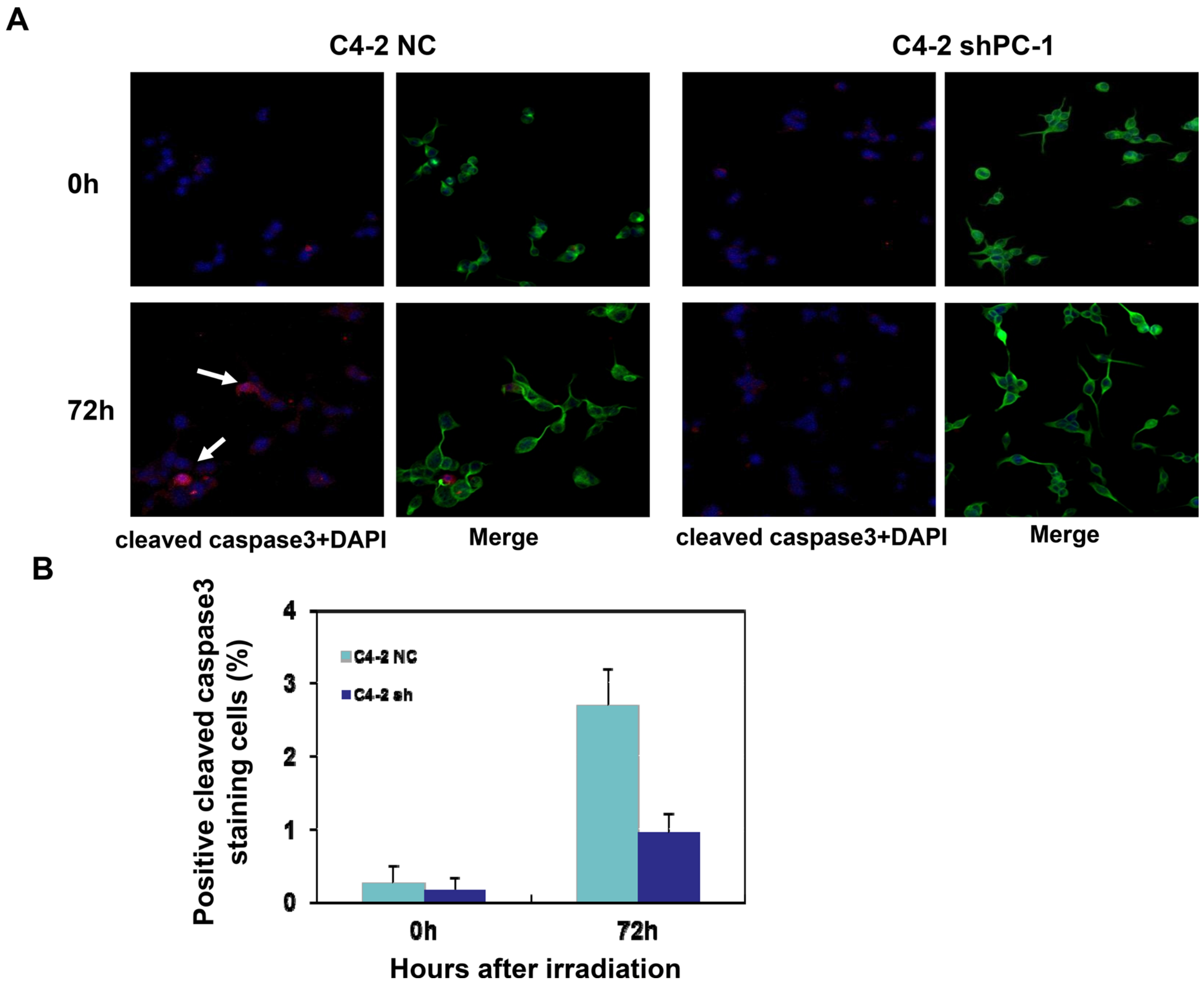

Supplementary Figure S2: C4-2 subline apoptosis after 4-Gy IR. A. C4-2 NC and C4-2 shPC-1 cells were IR or not and 3 days later stained with an antibody against caspase-3 and with DAPI (arrows, cleaved caspase-3-positive cells). **B.** Cleaved caspase-3 expression in C4-2 sublines.

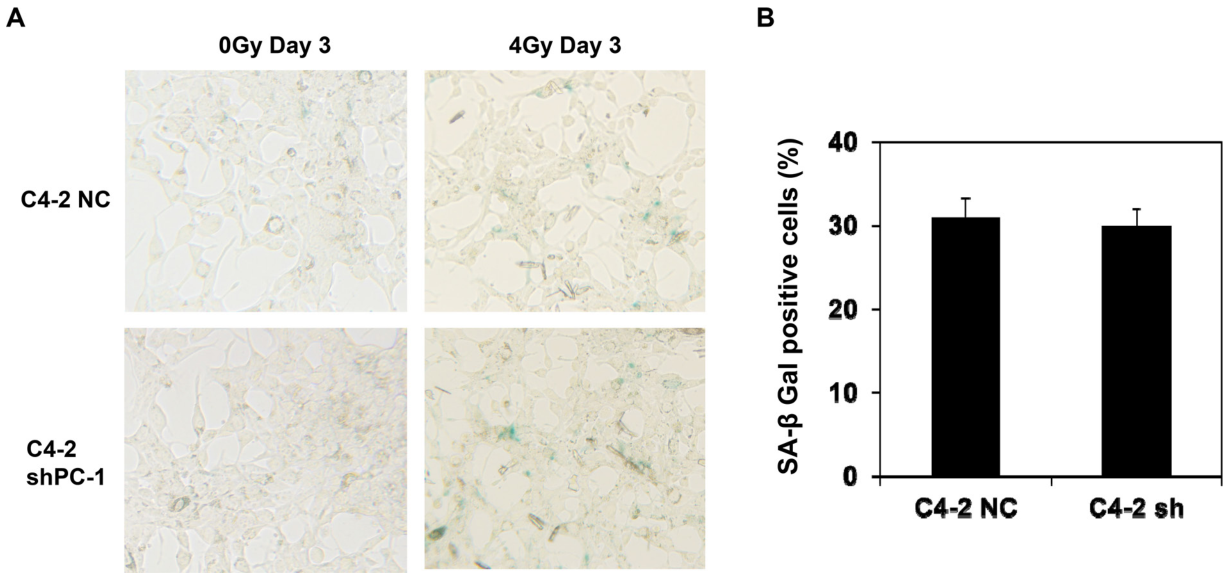

Supplementary Figure S3: Loss of PC-1/PrLZ did not change IR-induced senescence in prostate cancer cells. A. Representative image of SA-βGal activity after 72 h treatment. **B.** Quantitative analysis of senescent cells. Data are means ± SD of three independent experiments.
